# Activation of the intrarenal renin-angiotensin-system in murine polycystic kidney disease

**DOI:** 10.14814/phy2.12405

**Published:** 2015-05-21

**Authors:** Takamitsu Saigusa, Yujing Dang, Marlene A Bunni, May Y Amria, Stacy L Steele, Wayne R Fitzgibbon, P Darwin Bell

**Affiliations:** Division of Nephrology, Department of Medicine, Medical University of South Carolina, Charleston SC and Ralph Johnson VA Medical CenterCharleston, South Carolina

**Keywords:** ADPKD, hypertension, intratubular RAS, primary cilium

## Abstract

The mechanism for early hypertension in polycystic kidney disease (PKD) has not been elucidated. One potential pathway that may contribute to the elevation in blood pressure in PKD is the activation of the intrarenal renin-angiotensin-system (RAS). For example, it has been shown that kidney cyst and cystic fluid contains renin, angiotensin II (AngII), and angiotensinogen (Agt). Numerous studies suggest that ciliary dysfunction plays an important role in PKD pathogenesis. However, it is unknown whether the primary cilium affects the intrarenal RAS in PKD. The purpose of this study was to determine whether loss of cilia or polycystin 1 (PC1) increases intrarenal RAS in mouse model of PKD. Adult *Ift88* and *Pkd1* conditional floxed allele mice with or without cre were administered tamoxifen to induce global knockout of the gene. Three months after tamoxifen injection, kidney tissues were examined by histology, immunofluorescence, western blot, and mRNA to assess intrarenal RAS components. SV40 immortalized collecting duct cell lines from hypomorphic *Ift*88 mouse were used to assess intrarenal RAS components in collecting duct cells. Mice without cilia and PC1 demonstrated increased kidney cyst formation, systolic blood pressure, prorenin, and kidney and urinary angiotensinogen levels. Interestingly immunofluorescence study of the kidney revealed that the prorenin receptor was localized to the basolateral membrane of principal cells in cilia (−) but not in cilia (+) kidneys. Collecting duct cAMP responses to AngII administration was greater in cilia (−) vs. cilia (+) cells indicating enhanced intrarenal RAS activity in the absence of cilia. These data suggest that in the absence of cilia or PC1, there is an upregulation of intrarenal RAS components and activity, which may contribute to elevated blood pressure in PKD.

## Introduction

Polycystic kidney disease (PKD) is a group of inherited kidney disease characterized by formation of multiple kidney cysts that can eventually lead to kidney failure. In humans, loss of function in polycystin 1 (PC1) and polycystin 2 (PC2) cause autosomal dominant PKD (ADPKD) (Saigusa and Bell 2015), whereas loss of function in fibrocystin/polyductin causes autosomal recessive PKD (ARPKD) (Guay-Woodford et al. 2014). Early development of hypertension (HTN) is a hallmark of PKD and occurs in more than 60% of patients with ADPKD and more frequently with ARPKD (Chapman et al. 1990, 2010; Guay-Woodford et al. 2014). Although, the mechanism(s) that lead to the elevation in blood pressure may differ between ADPKD and ARPKD, they both have the activation of the renin-angiotensin-system (RAS) (Kennefick et al. 1999; Lawson et al. 2006). Activation of RAS may be due, at least in part, to cyst expansion resulting from compression of the renal vasculature leading to a stimulus for renin release. However, there is no consistent relationship between blood pressure and plasma renin activity or plasma aldosterone concentrations in humans with PKD (Kaplan et al. 1989; Chapman et al. 1990, 2010; Doulton et al. 2006). Another possible explanation is that intrarenal RAS instead of the systemic RAS maybe responsible for HTN. As discussed by Kobori et al. (2007), all components of RAS are normally located within the kidney, thereby leading to the generation of intrarenal AngII, which is regulated differently compared to systemic RAS. In PKD, renin, Agt, and AngII are produced by cysts, dilated tubules, and these components have been found to be present in cystic fluid (Graham and Lindop 1988; Torres et al. 1992; Loghman-Adham et al. 2004, 2005). Notably, in human cyst cells, Agt is expressed primarily in proximal tubule epithelial cells. Therefore proximal tubular Agt-generated AngII formation may contribute to the activation of intrarenal RAS, increased blood pressure and may stimulate renal epithelial cell proliferation /fibrosis which may lead to cyst expansion (Cao and Cooper 2001; Belibi and Edelstein 2010).

Another important factor in the pathogenesis of PKD is the primary cilium. It is well known that cystoproteins are located in cilia and, when mutated, result in altered cilia function leading to altered cell signaling and cyst formation (Lehman et al. 2008). For example, deleting IFT88, an intraflagellar transport protein in *Ift88* floxed allele in adult mice, results in stunted cilia, cilia dysfunction and slow development of cystic kidney disease resembling ARPKD (Davenport et al. 2007). Deletion of polycystin1 in adult *Pkd1* floxed allele mice leads to slow formation of kidney cysts resembling ADPKD (Piontek et al. 2004). Cilia dysfunction has also been linked to altered vascular structure/vascular responses (Torres et al. 2001; Kim et al. 2000), dysfunctional endothelial cell responsiveness to shear stress (Nauli et al. 2008), and altered Na^+^ handling at the collecting duct (Veizis et al. 2004; Olteanu et al. 2006) which may all affect systemic blood pressure and the RAS system. Although RAS is activated in PKD, it is unknown whether cilia dysfunction is involved in intrarenal RAS activation. Here we determined whether deleting primary cilia or polycystin1 (PC1) alters the RAS/intrarenal RAS using *Ift88* (ARPKD) *or Pkd1* (ADPKD) conditional knockout mouse (Piontek et al. 2004; Davenport et al. 2007) and an immortalized collecting duct cell line derived from a mouse that has an *Ift*88 hypomorphic gene deletion.

## Materials and Methods

### *Ift88 and Pkd1* conditional knockout mice

*Ift88*^*flox/flox*^ mouse was kindly provided by Dr. Bradley Yoder (UAB). Development of the *Ift88* floxed allele mice has been previously reported (Davenport et al. 2007). *Ift88*^*flox/flox* ^mouse^ ^have loxP sites flanking exon 4–6 of *Ift88. The Pkd1*^*flox/flox*^ mouse was kindly provided by Dr. Gregory Germino (NIH) (Piontek et al. 2004, 2007). *Pkd1*^*flox/flox*^ mice possess loxP sites on either side of exons 2–4 of *Pkd1*. *Ift88* and *Pkd1* conditional knockout mice were generated by crossbreeding *Ift88* ^*flox/flox*^ or *Pkd1*^*flox/flox*^ female mice with male mice that express the tamoxifen inducible systemic-cre with an actin promoter (CAGG-creER^™^) (Hayashi and McMahon 2002). Genotyping for both *Ift88* and *Pkd1* mice were performed by PCR using primers sequences as described previously (Piontek et al. 2004; Bell et al. 2011). Mice were maintained in accordance with the Institutional Animal Care and Use Committee regulations at the Medical University of South Carolina. Male and female mice at approximately 8–12 weeks of age were randomly selected for this study. To induce cre, tamoxifen (Sigma, St. Louis, MO) dissolved in corn oil (Sigma) was administered intraperitoneally (5 mg/20 g body weight) to *Ift88* ^*flox/flox*^ and *Pkd1* ^*flox/flox*^ mice with or without cre expression. Blood pressure (BP) was measured via tail cuff (Kent Scientific, Torrington, CT) or under anesthesia at 3 months after global gene knockout. Tail cuff systolic blood pressure data are from an average of 10–15 measurements. Since *Ift88*^*−/−*^ mice were too obese to fit in tail cuff blood pressure chambers, invasive arterial BP was measured as an alternative for *Ift88* mice. Mice were anesthetized with isoflurane and placed on a heated table to maintain body temperature at 37°C. The left femoral artery was cannulated and BP was measured with a Digi-Med BP analyzer system (Micro Med, Louisville, KY). Mice were euthanized with isoflurane overdose and kidney tissue was removed, preserved in 4% formalin or frozen.

### Measurement of creatinine, albumin, glucose, and urinary angiotensinogen

24 h urine samples were collected on ice via metabolic cages. Mouse plasma was collected via aorta puncture. Serum creatinine, urine creatinine, and albumin were measured using QuantiChrom assay kit (BioAssay Systems, Hayward CA). Fasting glucose was measured using a glucometer from blood samples obtained by tail stick. Urinary angiotensinogen was measured using an ELISA kit (Clontech, Mountain View CA) following the manufacturer's instructions.

### Cell culture

Studies were performed using a temperature-sensitive SV 40 immortalized collecting duct cell line derived from Oak Ridge Polycystic Kidney (Orpk) mouse model that is hypomorph for *Ift88* gene (Yoder et al. 2002). Cilia are absent or severely stunted in this cell line, which we designated as cilia (−). The *Ift88* gene was reintroduced into this cell line as a control and is designated as cilia (+). The cilia in these rescue cells have previously been shown to be phenotypically present and functional (Siroky et al. 2006a; Sas et al. 2011). Both cell lines were cultured in DMEM/F12 containing 5% FBS, 100 U/mL penicillin, 100 mg/mL streptomycin, 10 nmol/L 3,3′5-triiodo-thyronine, 50 nmol/L dexamethasone, 1.0 mg/mL insulin, 0.55 mg/mL human transferrin, 0.5 *μ*g/mL sodium selenite, 12 U/mL IFN-γ, and 500 *μ*L/L G418. Cells were cultured at the permissive temperature of 33°C in humidified air with 5% CO_2_ either on permeable filters (Transwell, 0.4-*μ*m pore size, polyester 24-mm^2^ membranes, Corning Costar, Cambridge, MA) or in 10-cm plastic culture dishes until confluent. To promote differentiation, cells were incubated at 39°C in the absence of IFN-*γ* for 4–5 days.

### Histologic analysis

For light microscopy, 5-*μ*m sections were cut from paraffin-embedded kidneys and stained with hematoxylin–eosin (H&E) and Masson trichrome. For immunofluorescence, paraffin-embedded kidney sections were deparaffinized in xylene and rehydrated in graded alcohols. Antigen retrieval was performed in boiling Tris-EDTA for 10 min. The slides were washed with Tris-buffered Saline (TBS) then blocked with background buster (NB306; Innovex Richmond, CA) for 1 h at room temperature. Rabbit polyclonal anti-renin antibody (1:200; 54371 AnaSpec, Fremont, CA) or anti-angiotensinogen (1:200; 28101A, Clontech) or anti-prorenin receptor: anti-ATP6IP2 (1:200; ab-40790 Abcam, Cambridge, MA) were added overnight at 4°C and then washed three times with TBS-0.1% Tween 20 (TBS-T), followed by the addition of Alexa 488-conjugated green fluorescent donkey anti-rabbit antibody (1:1000) incubated for 45 min at room temperature. After 5 washes with TBS-T, goat polyclonal anti-AQP2 antibody (1:100; SC-9882, Santa Cruz, Dallas, TX) was applied for 1 h at room temperature followed by Alexa 594-conjugated red fluorescent donkey anti-goat antibody (1:1000) for 1 h in room temperature. Hoechst (1:500) was added to the last wash in TBS at 1:500 and mounted with mounting medium. Slides were examined by confocal laser microscopy and images were all taken at fixed laser settings (Leica, Wetzlar, Germany).

### Cyst quantification

H&E-stained sections were used to determine cyst volume. At least 8–10 different kidney images at 5× were randomly taken from each of 90, 180, and 270° from the hilum to avoid field selection variation. Mean cystic area relative to total kidney section area was calculated using Image J (NIH, Bethesda, MD) and results are shown as percent.

### Western blot analysis

Mouse kidney tissue was homogenized for protein extraction. Cells were collected in ice-cold radio-immunoprecipitation assay buffer. Protease/phosphatase inhibitors (Thermo Scientific, Waltham, MA) were added to each sample and equal amounts of protein was resolved on a 10–20% SDS-PAGE and transferred to PVDF membranes (Life Technology, Grand Island, NY). The membrane was blocked with 5% nonfat milk followed by incubating with rabbit anti-renin antibody (1:1000) or anti-ATP6IP2 antibody (1:1000) or anti-angiotensinogen antibody (1:1000) overnight. After washing, the membrane was incubated with horseradish peroxidase (HRP)-conjugated secondary antibody (Millipore, Billerica, MA). Bands were visualized using chemiluminescence (ECL; Amersham International, Bucking Hamshire, UK).

### Real-time quantitative PCR

Mouse kidney tissue or collecting duct cells were homogenized at 4°C using a Biospec mini bead beater (Bartlesville, OK). Total RNA was isolated using an RNeasy mini kit (Qiagen, Valencia, CA). About 1 *μ*g of total RNA was used for cDNA synthesis using the RT^2^ first strand kit (Qiagen) and reverse-transcribed using RT^2^ SYBR Green MasterMix and the following primers, Agt (PPM04219E): 5′ GCTGAATGAGGCAGGAAGTG, 3′ GCAGCGAGGAC-CTTGTGTC, Ren1 (PPM33748B): 5′ GGCCAAGTTTGACGGTGTTC, 3′ ACAGAGAACACTTCCTCCTTTAGC, and GAPDH (PPM02946E): 5′ TATGACTCCACTCACG-GCAAATTC, 3′ ACATACTCAGCACCGGCCTC (Qiagen). GAPDH was used as a reference gene for normalization. Data are fold change relative to control from at least four separate determinations.

### Cyclic AMP assay

cAMP Enzyme Immunoassay Kit (Sigma) was used to measure cAMP levels in both cilia (−) and cilia (+) cells grown on permeable supports. Cells were treated with or without losartan (10 *μ*mol/L, Sigma) 15 min before applying apical Ang II (100 nmol/L, Sigma) or forskolin (20 *μ*mol/L). After 15 min, cells were lysed with 0.1 mol/L HCl for 10 min then scraped, collected, and centrifuged at 2100 *g* for 3 min. The supernatant (100 *μ*L) was incubated for 2 h with cAMP enzyme immunoassay antibody (50 *μ*L) and alkaline phosphatase conjugate (50 *μ*L). After washing, p-Nitrophenyl phosphate substrate was added and incubated at room temperature for 1 h. Then stop solution (trisodium phosphate) was added and optical density at 405 nm was measured (Spectramax M5 plate reader; Molecular Devices, Sunnyvale, CA). A Standard curve was constructed to calculate cAMP concentration from the biological samples and results were normalized by protein concentration.

### Statistical analysis

Results are shown as means ± SD or fold increase for mRNA. The significance of the results was determined by unpaired *t*-test or one-way ANOVA followed by Holm-Šídák test for post hoc comparison (GraphPad Prism 6.0, La Jolla, CA). A value of *P < 0.05 was considered to denote statistical significance and was marked with an asterisk in figures and tables.

## Results

### Kidney histology and mouse lines

**Figure 1:**
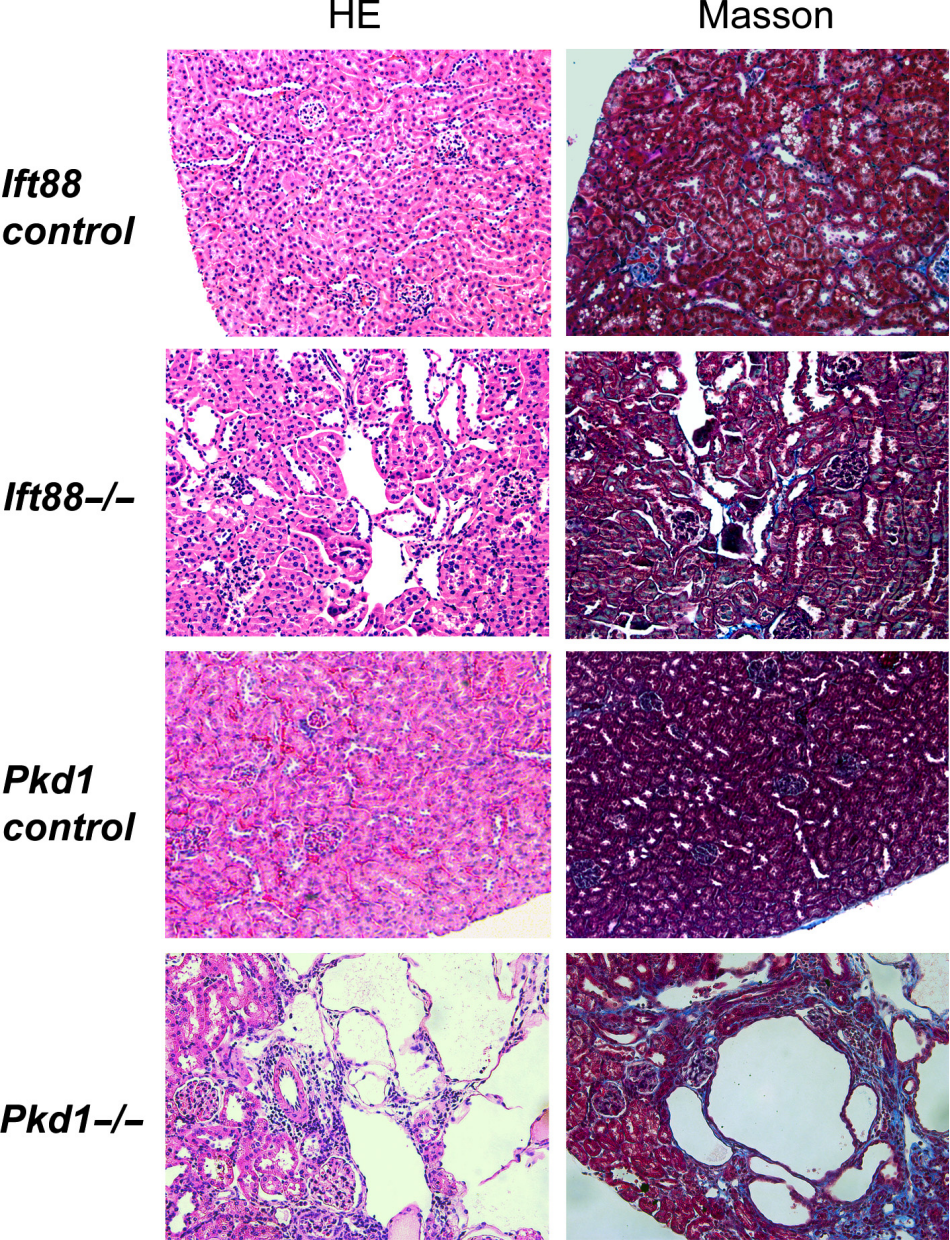
A representative image of the kidney from *Ift88*^*−/−*^*, Ift88 control*,* Pkd1*^*−/−*^*,* and *Pkd1 control* mice at 3 months after cre induction (H&E and Masson). There are focal kidney cysts seen in *Ift88*^*−/−*^ and much larger focal cysts in *Pkd1*^*−/−*^ mouse (*N* = 4). However, there were no cystic lesions seen in *Ift88* or *Pkd1* control kidneys. There was mild focal peri-cystic interstitial fibrosis (blue) seen in *Pkd1*^*−/−*^ mouse kidney by Masson Trichrome staining. However, there were no fibrotic changes in *Ift88*^*−/−*^ or control kidneys from both *Ift88*^*-*^ and *Pkd1* control mouse. Kidney sections are all shown at 20× magnification.

*Ift88*^*flox/flox*^ *(cilia) or Pkd1*^*flox/flox*^ mice that were cre-negative are designated as *Ift88 control* or *Pkd1 control* and, *Ift88*^*−/−*^ or *Pkd1*^*−/−*^ are cre-positive mice. Tamoxifen administration in cre-positive mice, has been shown to result in >80% deletion of cilia (Haycraft et al. 2007) and >50% efficiency for deletion of polycystin 1 (Piontek et al. 2007). Figure1 shows kidney histology from *Ift88*^*−/−*^, *Pkd1*^*−/−*^, *Ift88* control or *Pkd1* control mice, 3 months post-tamoxifen injection. H&E staining of kidneys from *Ift88*^*−/−*^ mice reveals significant development of focal kidney cysts primarily in the renal cortex compared to control (*Ift88*^*−/−*^vs. *Ift88 control* cyst ratio: 4.9 ± 0.9% vs. 0.9 ± 0.2%, *P* < 0.05, *N* = 4). Similarly, *Pkd1*^*−/−*^ mice developed significant focal kidney cysts compared to *Pkd1 control* mice (*Pkd1*^*−/−*^ vs. *Pkd1 control* cyst ratio: 11.5 ± 2.3 vs. 0.6 ± 0.1%, *P* < 0.05 *N* = 4). Masson trichrome staining demonstrates modest focal interstitial fibrosis surrounding kidney cysts (blue: Fig.1 right) in *Pkd1*^*−/−*^ mice. There were minimal to no tubular interstitial fibrosis seen in *Ift88*^*−/−*^, *Ift88 control,* and *Pkd1 control* mice. Table1 shows overall characteristics for both *Ift88*^*−/−*^ and *Pkd1*^*−/−*^ mice. *Ift88*^*−/−*^ mice become hyperphagic and increased body weight significantly compared to *Ift88 control* mice. Kidney size was not different between *Ift88*^*−/−*^ mice compared to *Ift88* control mice. However, *Pkd1*^*−/−*^ mice had larger kidneys (kidney weight to body weight ratio) compared to *Pkd1* control mice. Since *Ift88*^*−/−*^ mice were too large to fit in tail cuff BP chambers, arterial blood pressure was measured under isoflurane anesthesia, which revealed increased systolic blood pressure in *Ift88*^*−/−*^ mice compared to *Ift88 control* mice. Similarly, *Pkd1*^*−/−*^ mice had significantly higher systolic blood pressure measured by tail cuff compared to control mice. However, there were no differences in urine albumin to creatinine ratio, plasma creatinine, glucose, in the presence or absence of cilia or polycystin 1. These data demonstrate that *Ift88* and *Pkd1* knockout mice develop focal kidney cysts, and elevated systolic blood pressure at 3 months compared to control mice.

**Table 1:** Mouse demographics (3 months after cilia or PC1 loss)

	*Ift88* control	*Ift88* ^*−/−*^	*Pkd1* control	*Pkd1* ^*−/−*^
Body weight (g)	35.5 ± 3.7	46.1 ± 6.7*	30.1 ± 4.5	28.5 ± 3.1
Kidney weight (g)	0.24 ± 0.04	0.23 ± 0.03	0.20 ± 0.02	0.26 ± 0.03
Kidney weight/ Body weight (%)	6.8 ± 0.4	4.8 ± 0.3 ***	7.5 ± 0.2	9.7 ± 0.3 ***
Systolic blood pressure (mmHg)	87 ± 5	97 ± 5*	132 ± 5	171 ± 10*
Plasma creatinine (mg/dL)	0.13 ± 0.1	0.15 ± 0.1	0.11 ± 0.03	0.12 ± 0.06
Urine albumin to cr ratio (mg/gcr)	1.58 ± 0.1	1.26 ± 0.4	0.24 ± 0.01	0.20 ± 0.01
Plasma glucose (mg/dL)	259 ± 8	283 ± 13	N/A	N/A

Blood Pressure (Arterial: *Ift88* mice, Tail cuff: *Pkd1* mice) Values are means ± SD.

* *P* < 0.05

*** *P* < 0.0001 (*N* = 5).

### Prorenin is increased in *Ift88*^*−/−*^ compared to Ift88 control kidneys and in immortalized collecting duct cell lines

**Figure 2:**
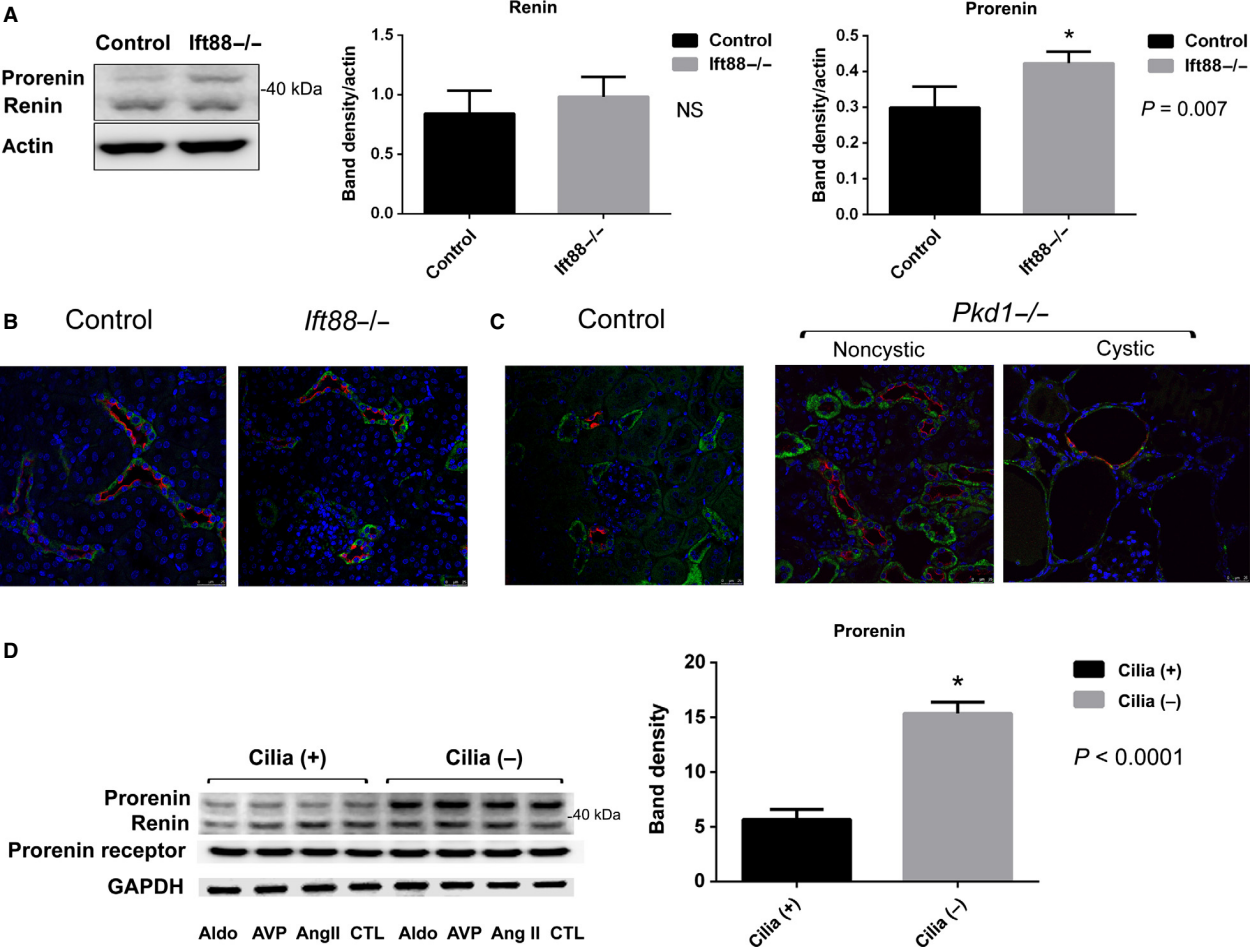
Renin and prorenin expression in kidney tissues and collecting duct cells. (A) Western blot analysis of whole kidney tissue revealed that renin (33kD) expression was not statistically different between *Ift88*^*−/−*^ and *Ift88 control*, but prorenin (42kD) was significantly higher in *Ift88*^*−/−*^ compared to *Ift88 control* (*P* = 0.007, *N* = 5). (B) Representative immunofluorescence (IF) image of the kidney from *Ift88*^*−/−*^*, Ift88 control* mice shows renin (green) was primarily localized in the collecting duct labeled with collecting duct marker AQP2 (red) in both *Ift88*^*−/−*^ and *Ift88 control* (green: renin, red: AQP2, blue: Hoecst (nuclei)). (C). Representative kidney IF image from *Pkd1*^*−/−*^ and *Pkd1* control mice. Renin (green) is primarily localized to the collecting duct and distal tubules but not in proximal tubules for both knockout and control mice. There are renin (green) in epithelia of collecting duct cyst lining cells (AQP2: red) as well as in other noncollecting duct cysts. (D). Western blot analysis of renin, prorenin, and prorenin receptor in collecting duct cell line reveals that cilia (−) but not cilia (+) cells have increased prorenin levels (*P* < 0.001, *n* = 5 GAPDH normalized). Stimulation with ang II (1 nmol/L) or DDAVP (10 nmol/L) or aldosterone (1 *μ*mol/L) did not increase renin, prorenin, or prorenin receptor levels.

RAS components in the kidney were assessed by immunofluorescence, RT-PCR, and western blot analysis. As shown in Fig.2A, there were no differences in kidney renin (33kD) levels, but kidney prorenin (42kD) levels were significantly increased in *Ift88*^*−/−*^ compared to *Ift88 control* mice by western blot analysis. There were no differences in kidney renin or prorenin levels in *Pkd1*^*−/−*^ and control mice (data not shown). Immunofluorescence (IF) demonstrated that renin (green) was primarily expressed in the collecting duct since it colocalized with aquaporin-2 (AQP2: red), a marker of principal cell, in both *Ift88*^−/−^ and *Ift88 control* kidneys (Fig.2B). IF images of kidneys from *Pkd1 control* mice also demonstrated that renin (green) is predominantly localized to the collecting duct (co-stained with AQP2) and in distal tubules but not in convoluted proximal tubules, similar to reports by others (Kobori et al. 2007) (Fig.2C). In *Pkd1* knockout mice, renin (green) was primarily expressed in noncystic collecting ducts, as well as along the cyst lining epithelia in both collecting duct (costained with AQP2) and non-AQP2 staining cysts; possibly of proximal tubular origin (Fig.2C). We next examined RAS components in the Orpk cilia (−) and (+) immortalized collecting duct cells culture grown on nonpermeable supports. As shown in Fig.2D, western blot analysis demonstrated increased prorenin (42kD) in cilia (−) compared to cilia (+) collecting duct cells consistent with the in vivo kidney tissue results. Since various hormones stimulate and regulate collecting duct transport, we tested whether AngII, vasopressin or aldosterone has any effect on the expression of renin/prorenin in these cell lines. There were no change in prorenin or renin levels after addition of ang II (1 nmol/L) or desmopressin (10 nmol/L) or aldosterone (1 *μ*mol/L) given 24 h and again 30 min before fixation. Renin and prorenin receptor protein expression were not statistically significant between cilia (+) and cilia (−) cells.

### Prorenin receptor is localized to the basolateral membrane in *Ift88*^*−/−*^ principal cells but not in Ift88 control

**Figure 3:**
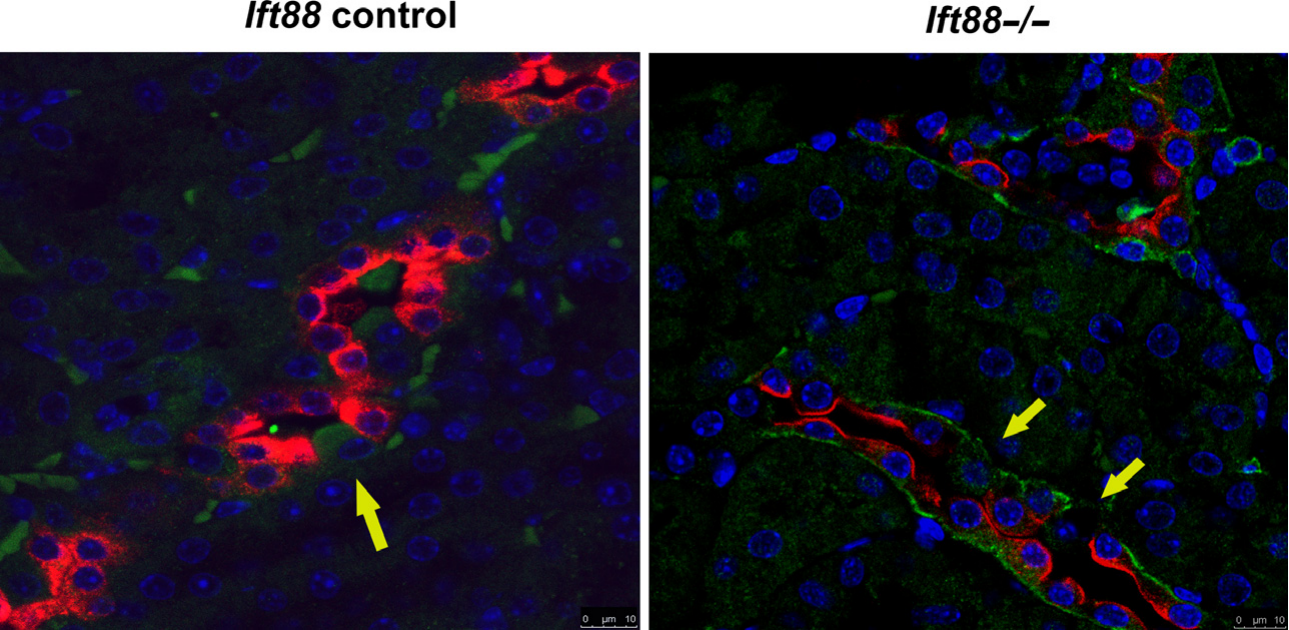
Representative IF image of prorenin receptor in *Ift88*^*−/−*^*, Ift88 control* kidney (80×). Prorenin receptor (green) is primarily localized to the intercalated cells (yellow arrow) in control kidneys. However, in *Ift88*^*−/−*^ kidney (right), prorenin receptor was localized to the basolateral membrane in principal cells but not in intercalated cells (AQP2: red). Note the yellow arrow, indicating intercalated cells, have negative basolateral staining in cilia (−) collecting duct.

Prorenin receptor is an accessory protein of vacuolar-type H^+^-ATPase and is generally localized in the intercalated cells in the collecting duct (Ludwig et al. 1998). Our studies confirmed, by IF, that the prorenin receptor (green) was primarily localized in intercalated cells (yellow arrow: lack of red AQP2 staining) in *Ift88 control* mouse kidneys (Fig.3: left). However, staining from *Ift88*^*−/−*^ mouse kidneys revealed that the prorenin receptor was predominantly localized to the basolateral membrane in the principal cells of the collecting duct (Fig.3: right). Interestingly, we did not observe basolateral prorenin receptor staining in the intercalated cells (yellow arrow), indicating that the prorenin receptor occurred only in the principal cells. Using IF, we found no differences in the localization of the prorenin receptor in cilia (+) and cilia (−) Orpk collecting duct cell line (data not shown). There were no differences in prorenin receptor protein and mRNA expression levels in kidneys between *Ift88*^*−/−*^ and *Ift88 control* mice (data not shown). Therefore, IF study suggests that loss of cilia results in localization of the prorenin receptor to the basolateral membrane of the principal cells.

### Loss of cilia or polycystin1 increases kidney angiotensinogen level

**Figure 4:**
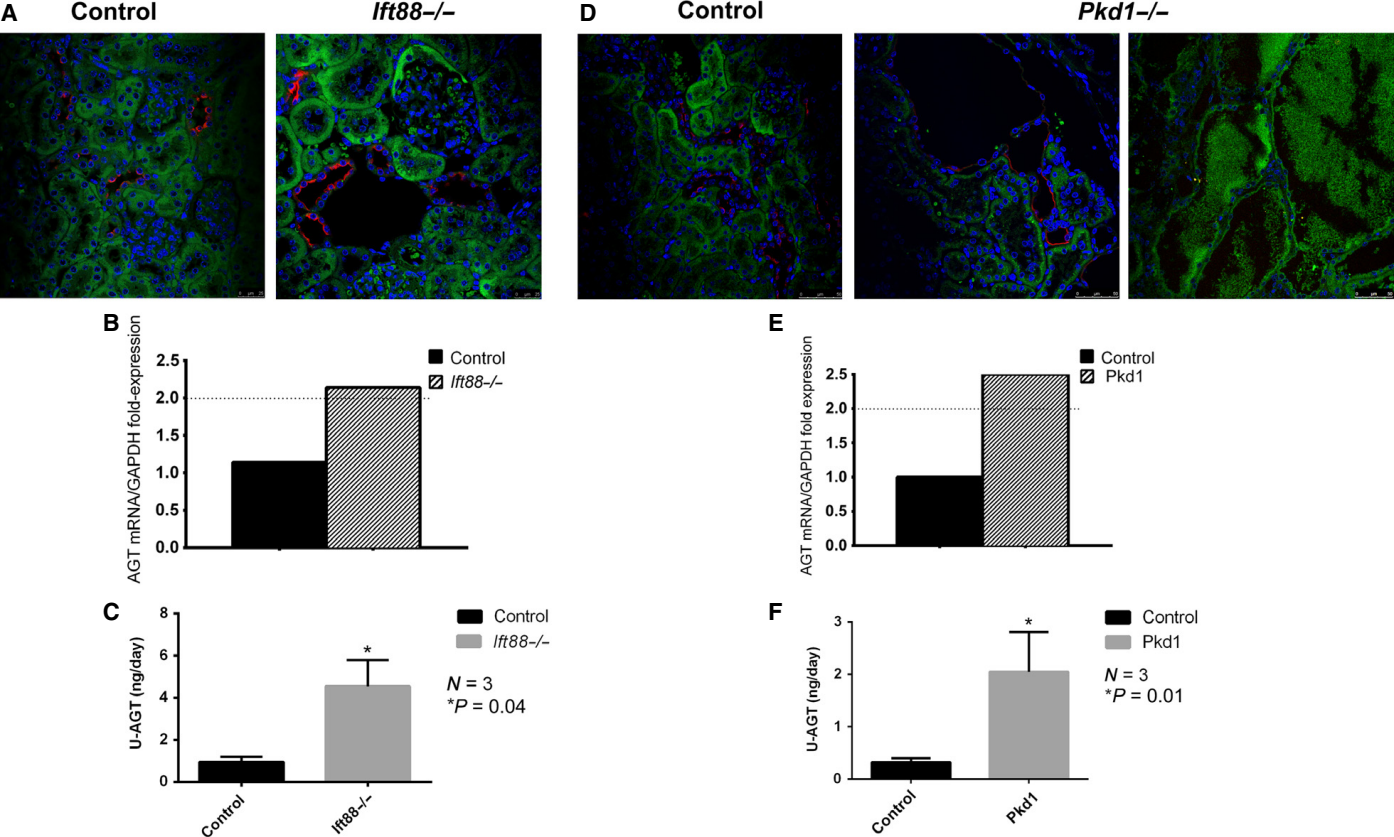
Angiotensinogen in the kidney. (A). Representative immunofluorescence image of Agt in *Ift88*^*−/−*^ and *Ift88 control* kidneys (magnification 60×). Agt (green) was primarily localized to the proximal tubules (co-stained with collecting duct marker AQP2: red) in both *Ift88*^*−/−*^ and *Ift88 control*. (B). Kidney Agt mRNA in *Ift88*^*−/−*^ kidneys shown as fold expression to control. There was a significant increase (over 2 fold) in cortical Agt mRNA levels in *Ift88*^*−/−*^ compared to control (normalized to GAPDH,* P* < 0.05). (C). Urinary Agt level measured by ELISA from *Ift88*^*−/−*^ and *Ift88 control* mice. There was a significant increase in urinary Agt level normalized by 24 h urine volume for *Ift88*^*−/−*^ compared to *Ift88 control* mice (*P* = 0.04, *N* = 3). (D). Representative immunofluorescence image of Agt in *Pkd1*^*−/−*^ and *Pkd1* control mice. Agt is localized at the proximal tubules in both *Pkd1*^*−/−*^ and *Pkd1 control* mice, negative for AQP2 staining. Agt is expressed along the cyst epithelia lining cells and in the cystic fluid in *Pkd1*^*−/−*^ (right). (E). Kidney Agt mRNA for *Pkd1*^*−/−*^ mice significantly increased compared to control (normalized to GAPDH,* P* < 0.05). (F). There was a significant increase in urinary Agt level in *Pkd1*^*−/−*^ mice compared to *Pkd1* control mice normalized by 24 h urine volume (*P* = 0.01, *N* = 3).

Angiotensinogen (Agt) is the primary substrate that is cleaved by renin to release angiotensin peptide. Studies have shown that there is local production or uptake of Agt in the proximal tubule epithelial cells in both normal and hypertensive animal models (Kobori et al. 2007). As shown in Fig.4A, Agt (green) was primarily localized in proximal tubules (which did not stain with AQP2: red) for both *Ift88*^*−/−*^ and *Ift88 control* (+) mice. Kidney Agt mRNA levels of *Ift88*^*−/−*^ kidney were increased compared to control (Fig.4B). We then examined urine Agt levels which is considered to reflect proximal tubular Agt production and is also a reflection of intrarenal RAS activity (Kobori et al. 2002). Our results demonstrate that 24 h urine Agt level was significantly higher in *Ift88*^*−/−*^ compared to *Ift88 control* mice (Fig.4C). Similar results were observed in *Pkd1*^*−/−*^ mice kidneys. IF for Agt (green) demonstrated a predominant localization in proximal tubules in *Pkd1 control* mice (Fig.4D). Agt is also expressed in proximal tubular cyst epithelia lining cells and in cystic fluid but there were no Agt in collecting duct cyst (positive for AQP2 staining, Fig.4D very right) in *Pkd1*^*−/−*^ mice. Kidney Agt mRNA levels of *Pkd1* knockout mice were significantly increased compared to control (Fig.4E). In addition, urinary Agt in *Pkd1*^*−/−*^ was increased compared to *Pkd1 control* mice urine. These results suggest that loss of cilia or *Pkd1* increases Agt mRNA and urinary Agt levels indicating increased intrarenal RAS.

### cAMP increases with apical angiotensin II stimulation and is inhibited by angiotensin-1-receptor antagonist in cilia (−) but not in cilia (+)cells

**Figure 5:**
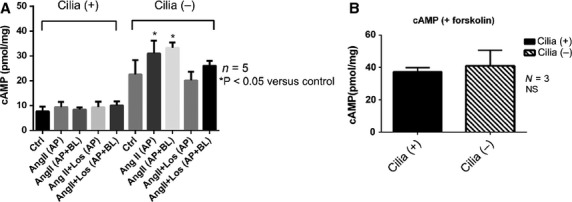
Cyclic AMP assay of cilia (−) and cilia (+) collecting duct cells. (A). Baseline cAMP level was higher in cilia (−) cells compared to cilia (+) cells (*P* < 0.001). Both apical (AP) and apical + basolateral (AP+BL) Ang II (100 nmol/L) significantly increased cAMP in cilia (−) but not in cilia (+) cells (*P* < 0.05). This increase was inhibited but losartan (10 *μ*mol/L). (B). Forskolin (10 *μ*mol/L) was added to the apical solution which resulted in similar increase in intracellular cAMP in both cell lines (NS, *n* = 3).

Cyclic AMP is a second messenger that induces cell proliferation, transcription, fluid secretion, and promotes cystogenesis in PKD (Belibi et al. 2004). Although RAS is elevated in PKD, it is unknown whether RAS activation directly contributes to intracellular cAMP levels in the PKD kidney. We therefore assessed intracellular cAMP levels in an immortalized PKD collecting duct cell line stimulated with AngII. Cells with cilia and without cilia were grown to confluency on permeable supports. At baseline cAMP was significantly elevated in cilia (−) compared to cilia (+) cells (Fig.5). Both cell lines were treated with Ang II (100 nmol/L) in the presence or absence of losartan (10 *μ*mol/L) for 15 min. Intracellular cAMP levels were measured using an immunoassay kit (Sigma) and results are shown in Fig.5. Apical or apical plus basolateral Ang II both significantly increased cAMP in cilia (−) and this increase was blunted by pretreatment with losartan (10 nmol/L). Interestingly, Ang II had no effect on cAMP levels in cilia (+) cells. Although forskolin (10 *μ*mol/L) apically produced equal increases in cAMP in both cilia (+) and cilia (−) (Fig.5B). These results indicate that there is an increase in cAMP in cilia (−) cells with Ang II stimulation.

## Discussion

Intrarenal RAS in the pathogenesis of hypertension in PKD has been proposed in both ADPKD and ARPKD (Loghman-Adham et al. 2004, 2005; Goto et al. 2010). For example, immunohistochemistry studies from patients with ADPKD and ARPKD demonstrated that cyst lining epithelial cells from cysts originating collecting duct have a high expression of renin and AT1-receptors while Agt is primarily expressed in cysts that are derived from proximal tubules. We found that loss of cilia or polycystin 1 results in increased kidney Agt levels by RT-PCR at 3 months post-cre induction. In addition, urinary Agt levels, a marker for intrarenal RAS activity were both elevated in *Ift88*^*−/−*^ and *Pkd1*^*−/−*^ mice but not in control mice. IF studies revealed that Agt is primarily localized at the proximal tubules with loss of either cilia or Pkd1 which is consistent with reports in animal models of hypertension and/or PKD (Kobori et al. 2007; Loghman-Adham et al. 2004, 2005). Interestingly, some distal/collecting duct cyst from Pkd1 mouse had Agt staining along the cyst lining epithelia. These results suggest that loss of cilia or *Pkd1* augments kidney Agt production, predominantly in the proximal tubule but Agt may also extend to epithelial lining of cyst derived from distal/collecting duct.

Although loss of cilia did not alter renin levels, there was a significant increase in kidney prorenin levels in *Ift88*^*−/−*^ compared to *Ift88 control* mouse. This finding was also consistent with cell culture studies, which demonstrated a higher prorenin protein expression in cilia (−) compared to cilia (+) collecting duct cells. Prorenin is a precursor of renin but its function has not been fully elucidated (Jan Danser et al. 2007). In general, chronic stimulation of RAS increases the conversion of prorenin to renin thereby decreasing the circulating prorenin levels (Jan Danser et al. 2007). It has been reported that transgenic mouse that overexpresses prorenin results in hypertension due to generation of AngII (Mercure et al. 2009). Therefore, high prorenin levels per se may contribute to hypertension. However, plasma prorenin levels in diabetic patient are reported to be also high (Luetscher et al. 1985) and may play a role in renal Ang II generation (Stankovic et al. 2006). In addition, intrarenal prorenin levels are elevated in the collecting duct from both diabetic and hypertensive rats (Prieto-Carrasquero et al. 2005; Kang et al. 2008; Liu et al. 2011). It is noteworthy that *Ift88*^*−/−*^ mice become hyperphagic and obese. However, at least at 3 months, plasma glucose levels were comparable to control mice in this study. Whether loss of cilia per se or hyperphagy/obesity contributes to increased prorenin production needs further study. We acknowledge that obesity may have influenced the development of hypertension, possibly by a mechanism distinct from that mediated by cystogenetic pathways. One interesting result was the localization of the prorenin receptor in the absence of cilia. Typically, the prorenin receptor is localized in intercalated cells since it serves as an accessory protein of the vacuolar-type H^+^-ATPase (Ludwig et al. 1998). However, in the absence of cilia, the prorenin receptor was primarily expressed and localized to the basolateral membrane of principal cells. In PKD, there is a disruption in apical-basolateral polarity in renal epithelia; for example, EGFR (Du and Wilson 1995; Coaxum et al. 2014), vasopressin-2 receptor (Saigusa et al. 2012) and Na^+^:K^+^ATPase (Wilson et al. 2000) have been reported to be expressed at the apical membrane. It has been suggested that in PKD, mislocalization of receptors may contribute to altered cell signaling, hyperproliferation of cyst-lining epithelia, and the secretory phenotype contributing to cyst expansion. However, at the present time, it is unclear why prorenin receptor was expressed in principal cells. It is noteworthy that increased prorenin and altered localization of the prorenin receptor were observed only when cilia was absent but not by loss of polycystin 1. Since cysts arise mainly from the collecting duct in ARPKD (Siroky and Guay-Woodford 2006b), it is interesting to determine whether increased prorenin stimulates the altered localized prorenin receptor leading to the activation of the intrarenal RAS at the collecting duct. Administrating prorenin peptide with or without a prorenin receptor inhibitor may benefit to see if prorenin activity is important in the pathology of the cilia knockout model.

In collecting duct cell culture studies, Ang II simulation increased cAMP levels in cilia (−) compared to cilia (+) cells and this response was blunted with AT1 receptor blocker, losartan. This difference in response was confirmed by the demonstration that addition of forskolin increased intracellular cAMP levels in both cell lines. Although there are conflicting results (Burns et al. 1996), recent studies have demonstrated that Ang II via the AT1R increases cAMP in the collecting duct (Wong and Tsui 2003). Li et al. (2011) have demonstrated that Ang II increased AQP2 expression in an immortalized mouse renal collecting duct principal cell (mpkCCD_cl4_) line through activation of cAMP, PKA, and calmodulin pathways, suggesting a cross talk between the V2R and AT1R. We have previously reported, in an in vitro study, that loss of cilia resulted in mislocalized V2R to the apical membrane leading to enhanced salt and water absorption in the collecting duct (Saigusa et al. 2012). If there is a cross talk between AT1R and V2R in PKD, then this would suggest that the activation of the cAMP-PKA pathway by intrarenal RAS, may lead to enhanced salt and water reabsorption in noncystic collecting duct cells thereby leading to elevated blood pressure in PKD. Since elevated cAMP is seen in PKD and is a strong contributor to cyst expansion (Belibi et al. 2004), suppressing the intrarenal RAS may not only lead to better BP control, but slow cyst expansion in PKD. We acknowledge that cell culture experiments were not conducted under conditions mimicking tubular flow.

There are several limitations in this study. Unlike human ARPKD, which typically results in severe kidney phenotype, *Ift88*^*−/−*^ mouse revealed only mild focal kidney cysts. Also, two different BP measurement methods were used between *Ift88* and *Pkd1* mice due to technical constraints. Utilizing telemetry blood pressure monitoring in PKD mouse may help to further resolve the issue of blood pressure regulation in PKD.

In summary, the results of this study suggest that a loss of cilia or polycystin 1 results in upregulation of the intrarenal RAS system which may contribute to hypertension that is found with increased frequency in PKD.
